# Detection of Epithelial Cell Adhesion Molecule in Feline Normal and Tumor Cell Lines and Tissues With Selected Commercial Anti-human EpCAM Antibodies

**DOI:** 10.3389/fvets.2021.622189

**Published:** 2021-02-04

**Authors:** Christa Y. Heyward, Lynn Dong, Hayk Shakhzadyan, Christopher Wan, Tracy Stokol

**Affiliations:** ^1^Department of Population Medicine and Diagnostic Sciences, College of Veterinary Medicine, Cornell University, Ithaca, NY, United States; ^2^Department of Biomedical Sciences, College of Veterinary Medicine, Cornell University, Ithaca, NY, United States

**Keywords:** cat, cancer, immunohistochemistry, flow cytometry, circulating tumor cells, mammary carcinoma, TROP-1/Ep-CAM, squamous cell carcinoma

## Abstract

Epithelial cell adhesion molecule (EpCAM) is a transmembrane protein expressed at intercellular junctions in epithelial cells. As an epithelial biomarker, it used for immunologic-based capture of epithelial-derived circulating tumor cells (CTCs) in human patients with different carcinomas. EpCAM expression has not been described in normal or neoplastic epithelial tissues in cats. Our goal was to find a commercial antibody that recognizes surface EpCAM expression for CTC detection. We tested two anti-human EpCAM antibodies, designated for use with flow cytometry, for detection of surface EpCAM expression on feline cell lines derived from normal mammary and renal epithelia and mammary and oropharyngeal squamous cell carcinomas in cats. Only one of the antibodies, a goat polyclonal antibody, labeled normal and neoplastic feline mammary epithelial cells and oropharyngeal squamous cell carcinoma cells; no labeling was observed for normal feline kidney epithelial cells. At low dilution, this antibody immunohistochemically stained the intercellular junctions of normal pancreatic, intestinal and mammary epithelium, as well as neoplastic mammary epithelium in feline tissues; however, oral mucosa, skin, and an oropharyngeal squamous cell carcinoma showed no positive immunostaining. The antibody only weakly bound feline squamous cell carcinoma cell lines under static adhesion. Our results indicate that EpCAM is expressed in specific epithelia in cats but is variably expressed in feline mammary tumors and oropharyngeal squamous cell carcinoma. A higher avidity cross-reactive or feline-specific antibody will be required to further investigate EpCAM expression in normal and neoplastic feline tissue or for detecting CTCs in the blood of tumor-bearing cats.

## Introduction

Blood-based “liquid biopsies” are becoming more prevalent in clinical diagnostic medicine because they can be readily performed and are minimally invasive, making them ideal for detection and monitoring of disease. Biomarkers used in liquid biopsies in humans include circulating tumor cells (CTCs), cell-free nucleic acids (DNA, RNA, microRNA), and cell-derived proteins, exosomes, lipids, and metabolic products (1). Detection and quantification of CTCs is being increasingly used as a diagnostic and prognostic marker in human patients with tumors, particularly those of epithelial origin (2–6). Most techniques used for identification of CTCs rely upon the immunologic detection of lineage-associated markers. One such marker for epithelial tumors is epithelial cell adhesion molecule (EpCAM), also known as epithelial glycoprotein 2 (EGP-2), epithelial specific antigen (ESA), GA733-2, 17-1A, HEA125, MK-1, KSA, Trop-1, tumor-associated calcium signal transducer 1 (TACSTD1) and CD326 (7, 8). EpCAM is a 39–42 kDa transmembrane glycoprotein expressed on the cell membranes of many epithelial, but not mesenchymal or neuroendocrine, tissues (9–11). EpCAM is also considered a marker of carcinogenesis, because it is over-expressed in many tumors of epithelial origin, even tumors arising from tissue which normally lack expression of the protein, such as squamous cell carcinoma (7–12). EpCAM plays a role in cell migration, adhesion, proliferation, differentiation and signaling in tumors (7, 8, 13). The fact that EpCAM expression is limited to epithelial cells makes it a good candidate for use as an epithelial-derived CTC marker, because human blood leukocytes typically lack EpCAM expression (14). Numerous studies have shown that EpCAM-positive cells can be detected in the circulation of human patients with various carcinomas and those patients with high numbers of CTCs have lower overall survival (4, 5, 15–17). Indeed, analyzers have been built for the specific purpose of detecting EpCAM-positive CTCs (e.g., CellSearch®) (5, 18).

Epithelial tumors are one of the most common tumor types affecting cats and are usually malignant. Primary sites of tumorigenesis in cats include the mammary gland, the gastrointestinal and respiratory tracts, and the skin (19). To our knowledge, EpCAM expression has not been evaluated on feline tumors. Due to the lack of anti-feline EpCAM antibodies, our objective was to test commercially available antibodies raised against human EpCAM for their ability to detect the protein in feline tissues and cell lines. Our goal was to find an antibody that could be used for detection of EpCAM on the surface of intact feline epithelial cells for possible future use as a biomarker of epithelial-derived CTCs in cats. Identifying a commercially available antibody with cross-reactivity to feline EpCAM would eliminate the need to produce feline-specific antibodies. For surface detection of EpCAM, we used flow cytometric analysis on cell lines derived from normal mammary and renal epithelium, mammary tumors and oropharyngeal squamous cell carcinoma. Antibodies that positively stained feline epithelial cells in flow cytometric experiments were verified by immunohistochemical staining of a feline tissue array and normal and neoplastic feline mammary and oropharyngeal tissue. We also determined if any cross-reactive antibodies could bind feline tumor cells under static assay conditions, reasoning that this would be the first requisite step to show the antibody could be used in future assays for detecting epithelial-derived CTCs in blood or body cavity samples (so-called “liquid biopsies”) from cats.

## Materials and Methods

### Sequence Identity

We analyzed the protein sequence identity between human (NCBI accession # NP_002345.2, UniProt P16422) and feline (NCBI accession # XP_019682709.1, UniProt M3WIV4) EpCAM using NCBI BLAST, UniPROT and Jalview sequence comparison tools (20–22).

### Antibodies

For surface EpCAM expression on intact cells, we tested two anti-human EpCAM antibodies, a rabbit monoclonal and a goat polyclonal antibody, for reactivity with human and feline cell lines using flow cytometry (Table 1). These antibodies were chosen because they were designated for flow cytometric use by the manufacturer. Rabbit serum (IMGENEX, Novus biological, a Bio-Techne Company Centennial, CO) or goat gamma globulin (γ-globulin; Jackson ImmunoResearch Laboratories, Inc, West Grove, PA) were used as negative controls for the relevant antibodies.

**Table 1:** Anti-human EpCAM antibodies tested for their ability to detect the protein on normal feline mammary and renal epithelial cell lines and neoplastic feline mammary epithelial and oral squamous cell carcinoma cell lines using flow cytometry.

**EpCAM** ** Antibody**	**Company**	**Clone**	**Catalog #**	**Immunogen**	**Source**
R&D EpCAM	R&D Systems, Minneapolis, MN	NA	AF960	Extracellular domain, Gln-24-Lys265	Goat polyclonal
SB EpCAM	Sino Biologicals, Wayne, PA	28	10694-R028	Recombinant EpCAM	Rabbit monoclonal

*These antibodies were designated for flow cytometric use by the manufacturer*.

### Cell Lines and Media

All reagents were from Corning (Corning, NY) or Sigma-Aldrich (St Louis, MO), unless otherwise specified. Human mammary carcinoma cell lines, MCF-7 and MDA-MB-231, were used as positive controls for EpCAM expression (11, 23, 24). MCF-7 cells were grown in Minimal Essential Media with 10% fetal bovine serum (FBS, Atlanta Biologicals, Flowery Branch, GA), 1% L-glutamate (Lonza, Salisbury, MD), 1% sodium pyruvate (Lonza), 2% sodium bicarbonate, and 0.1% human insulin and used between passages (p) 4 and 27. MDA-MB-231 cells (p70–88) were grown in complete medium, consisting of Dulbecco's Modified Eagle Medium (DMEM) with 10% FBS and 1% penicillin, and streptomycin. Feline mammary carcinoma cell lines, CAT-MT (RRID:CVCL_T987, p19–44) (25) and K12-72.1 (RRID:CVCL_IX41, p13–39) (26) (from Dr. Gerlinde van de Walle, Cornell University), and oropharyngeal squamous cell carcinoma cell lines, SCCF-2 (p6–29) and SCCF-3 (p2–17) (27) (from Dr. Joseph Wakshlag, Cornell University), were also grown in complete medium. Feline mammary epithelial cells (FMEC, p36–69, from Dr. John Parker, Cornell University) (28) and Norden Laboratory feline kidney (NLFK) cells (p41–93, from Dr. Colin Parrish, Cornell University) (29) were used as non-tumorigenic epithelial control cell lines. C10 injection site sarcoma cells (p43–66, from Dr. Kelly Hume, Cornell University) (30) were used as a non-epithelial tumor cell control. FMEC cells were grown in a 50/50 v/v mixture of DMEM and F12K (Ham's F-12K Nutrient Mixture, Kaighn's Modification with L-glutamine) with 10% FBS, 1% penicillin, and streptomycin, 1% non-essential amino acids (NEAA), and 10 ng/mL endothelial mitogen factor (MP Biomedicals, Fisher Scientific, Rockford, IL). The NLFK cells were grown in a 50/50 v/v mixture of L-15 and McCoy's A5 Media with 10% FBS and 0.1% gentamicin (Gibco/Life Technologies, a division of ThermoScientific, Waltham, MA). The C10 cell line was grown in DMEM with 10% FBS, 1% L-glutamine, 1% NEAA, and 1% penicillin and streptomycin. Cells were detached using 0.1% trypsin-EDTA for use. Cell viability was >90% based on trypan blue exclusion.

### Flow Cytometric Analysis

Both antibodies were tested for their ability to recognize EpCAM on the surface of the human positive control cell lines, MCF-7 and MDA-MB-231. Antibodies (1:50 dilution of a 0.2 mg/ml stock or 4 μg/ml final concentration) or negative control rabbit serum or goat γ-globulin (at an equivalent concentration to the primary antibody) were incubated with cells for approximately 30 min and then washed three times with phosphate-buffered saline (PBS) and 1% bovine serum albumin (BSA). Cells were subsequently incubated with the appropriate FITC-conjugated secondary antibody (1:200 dilution, Invitrogen, a division of ThermoScientific), incubated for an additional 30 min, and washed three times in PBS-BSA. After washing, cells were resuspended in 300–500 μl of PBS and data was collected on a FACS Calibur (BD Biosciences; Franklin Lakes, NJ). Data was analyzed using Flowjo software (BD Biosciences; Ashland, OR). Once EpCAM expression on the human positive control breast cancer cell lines was confirmed for each antibody, the antibodies were then evaluated for binding to the feline cell lines using flow cytometry. For competition experiments, 5 × 10^5^ cells were incubated with 0.5 and 1.5 μg of recombinant human EpCAM (R&D Systems), then labeled with the primary antibody or negative control, as above.

### Immunohistochemical Staining of Feline Tissues

To test whether the antibody that positively labeled feline normal and neoplastic epithelial cells on flow cytometric analysis (the goat polyclonal R&D EpCAM) could detect a protein with expected membrane-associated expression in normal and neoplastic feline tissue, we performed IHC staining on formalin-fixed paraffin-embedded tissue. This staining was initially performed on an array prepared from tissues from a healthy cat by the Histology Laboratory in the Animal Health Diagnostic Center at Cornell University. The array contained the following tissues: liver, renal cortex, oral mucocutaneous junction, pancreas, stomach (fundus), duodenum (distal to papilla), jejunum, colon, lung, adrenal gland, thyroid gland, pituitary gland (pars nervosa), brain (frontal lobe), testes, outer aorta, heart, mandibular and mesenteric lymph node, tonsil, spleen, thymus, and skeletal and smooth muscle. Additional sections of normal feline colon were used to optimize the immunostaining procedure. Immunostaining for EpCAM was then performed on two feline mammary carcinomas, one of which had adjacent, non-neoplastic mammary epithelium as an internal control and normal cutaneous epithelium, and an oropharyngeal squamous cell carcinoma with normal oral tissue obtained from a healthy cat as a negative control. The mammary carcinomas were typed as a grade II tubulopapillary carcinoma and a grade III tubular carcinoma (31). The mammary carcinomas were archived samples in the Histology Laboratory, whereas the oropharyngeal squamous cell carcinoma was obtained from prospectively collected tissue that was placed in the Cornell University Biobank. The latter protocol was approved by the Institutional Animal Use and Care Committee at Cornell University (#2005-0151). For both initial and optimized procedures, 4 μm–thick sections of formalin-fixed, paraffin-embedded sections were deparaffinized in xylene and rehydrated with graded ethanol, followed by antigen retrieval steaming for 20 min in either Tris-EDTA (pH 9.0) (initial protocol) or sodium citrate (10 mM, pH 6.0) (optimized protocol). Endogenous peroxidase activity was quenched with 0.3% hydrogen peroxide in distilled water for 10 min. In the optimized procedure, non-specific staining was blocked with a mixture of 10% rabbit serum and 2 × casein for 1 h at room temperature. Immunostaining was then performed using the ImmPRESS HRP Anti-Rabbit Ig (Peroxidase) Polymer Detection Kit (Vector Laboratories, Burlingame, CA) according to kit instructions. The latter kit used 3,3′ diaminobenzidine as a chromogen. In the initial procedure, sections were incubated with various dilutions of the goat polyclonal R&D EpCAM antibody overnight at 4°C (1:10–1:200). In the optimized procedure, the antibody was incubated at a 1:10 dilution for 3 h at room temperature, followed by 20 h at 4°C. Negative controls were run in parallel by replacing the primary antibody with goat immunoglobulin G (IgG) at an equivalent final concentration. After washing three times in PBS with 0.05% Tween 20, the sections were incubated with biotinylated rabbit anti-goat IgG (1:200, Vector Laboratories, Burlingham, CA) for 1 h at room temp, followed by streptavidin-horseradish peroxidase conjugates (Ready to use, Vector Laboratories) for 20 min at room temperature. For some tumors with the optimized procedure, Nova Red (Vector Laboratories,) was used as an alternative chromogen to visualize antigen localization and sections were lightly counterstained with hematoxylin. Stained sections were examined with an Olympus AX 70 compound microscope, equipped with MicroFire camera and PictureFrame for image processing and capture (Optronics, Goleta, CA) or an upright microscope (BX40, Olympus Corporation, Life Science Solutions, Center Valley, PA), equipped with a digital camera (Olympus, model UC90), using cellSens software (standard 1.18, Olympus).

### Static Adhesion Assay

To determine if the goat polyclonal R&D EpCAM antibody could capture feline tumor cells under static conditions, we used a modification of our previously described assay (32). For this assay, we compared the feline oropharyngeal squamous cell carcinoma cell lines (SCCF-2, SCCF-3), with the MCF-7 human breast carcinoma and the C10 feline injection site sarcoma cell lines as positive and negative cell controls, respectively. In each experiment, duplicate coverslips were coated for 1 h at 37°C with the goat polyclonal R&D EpCAM antibody (1:50 final dilution, 4 μg/ml), using equivalent concentrations of goat γ-globulin as a negative control and fibronectin (20 mg/ml, EMD Millipore, Burlington, MA) as an integrin-mediated binding control. The rabbit monoclonal SB EpCAM antibody (1:50) was used as a positive antibody-binding control for the human MCF-7 cell line. After incubation, coverslips were washed three times with sterile PBS, and then blocked by incubation with 1% PBS-BSA for 30 min at 37°C. Coverslips were then seeded with 5 × 10^4^ cells and incubated at 37°C for 1 h to allow for attachment. The coverslips were subsequently washed before fixing the cells with 4% paraformaldehyde. After additional washes, the coverslips with adherent cells were mounted to glass slides with Prolong™ Gold antifade mounting media, which includes 4′-,6′- diamidino-2-phenylindole for nuclear staining (Thermo Fisher Scientific). Ten random fields were then captured using an Axio Imager M1 microscope (Zeiss, Thornwood, NY, USA). A blinded investigator then reviewed the captured saved images and counted DAPI-stained nuclei of adherent cells. Each cell line was tested in at least two separate experiments.

### Statistical Analysis

Data was analyzed with GraphPad Prism software (San Diego, CA) and displayed as mean ± standard deviation (SD) if Gaussian or median and range if non-Gaussian, as assessed by a Shapiro-Wilk normality test. Median fluorescence intensity (MFI) for all flow cytometry samples was obtained using Flowjo analysis software (BD; Ashland, OR) and the MFI of the γ-globulin control subtracted to obtain the delta MFI. For the static adhesion assay, the median number of cells attached under each condition was compared by Kruskal Wallis Analysis of Variance and a Dunn's multiple comparison test. Significance was set at *p* < 0.05.

## Results

### Sequence Alignment

**Figure 1:**
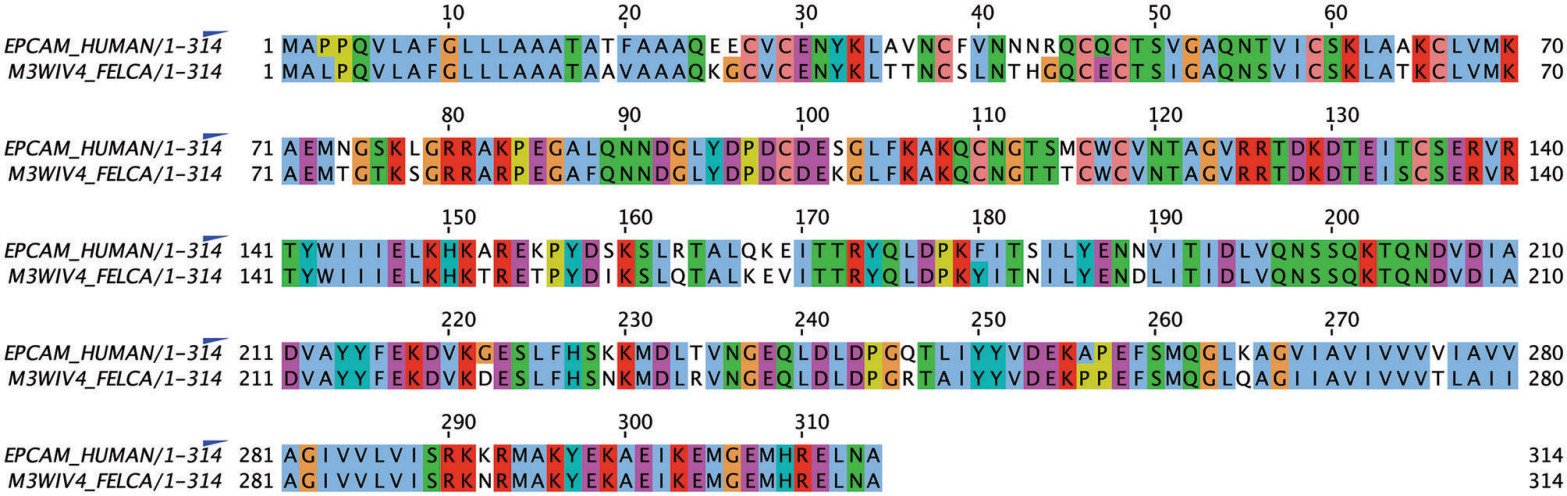
Alignment of human and feline EpCAM amino acid sequences using UniProt sequences for human (P16422) and feline (M3WIV4) EpCAM sequences (Jalview, version 2.11.1.0). Similar colored boxes show sequence alignment.

Alignment of the published protein sequence of human and feline EpCAM revealed 84% identity (Figure 1).

### Antibody Labeling of Human Breast Carcinoma Cell Lines and Normal and Neoplastic Feline Cell Lines With Flow Cytometric Analysis

**Figure 2:**
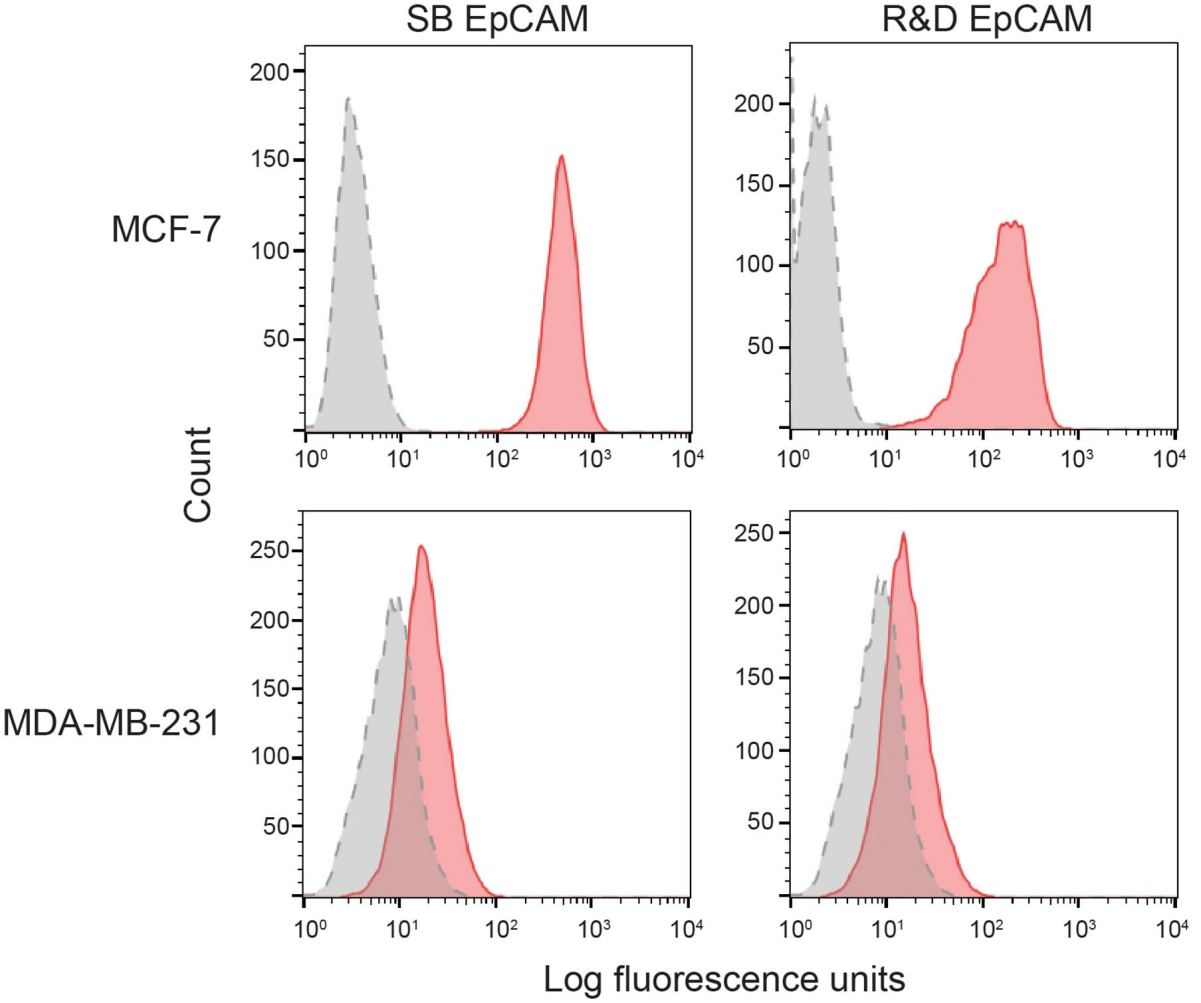
Binding of the two selected anti-human EpCAM antibodies (SB EpCAM, R&D EpCAM) to human breast carcinoma cell lines, MCF-7 and MDA-MB-231, by flow cytometry. Representative frequency distribution curves of logarithmic fluorescent intensity for each cell line are shown (*n* = 3 independent experiments) (EpCAM antibodies: pink solid curves; rabbit serum or goat gamma globulin controls: gray solid curves with dotted line).

**Figure 3:**
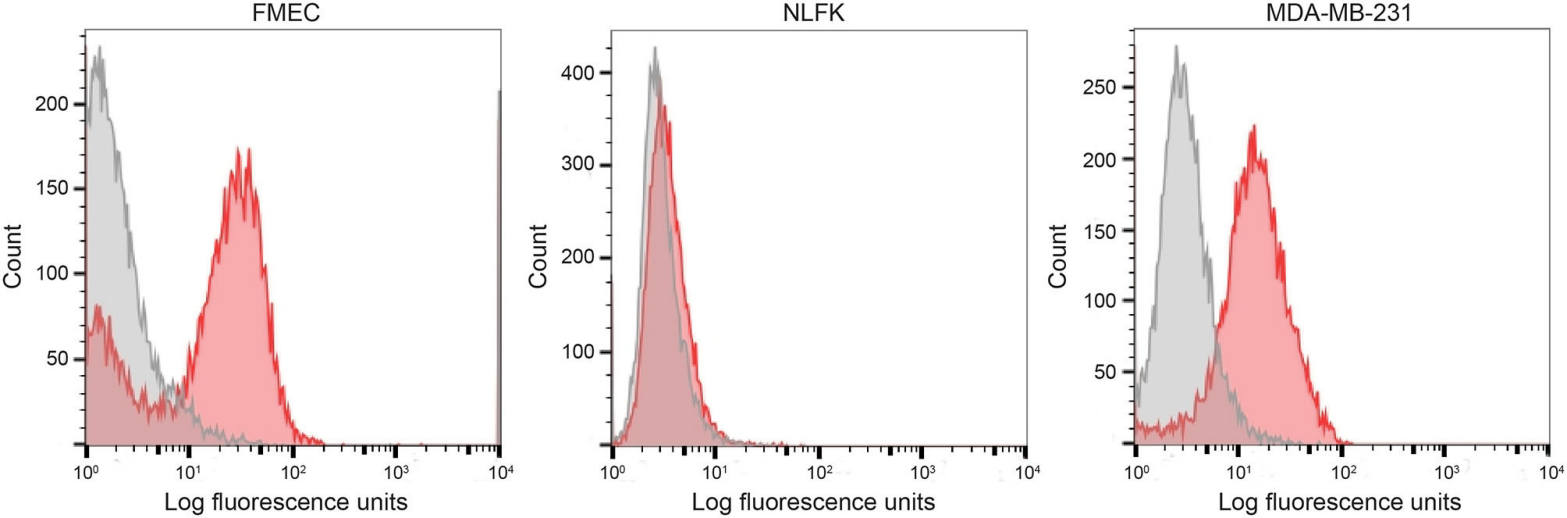
Labeling of feline normal mammary (FMEC) and renal (NLFK) epithelial cell lines with a goat polyclonal anti-human EpCAM antibody (R&D EpCAM) with flow cytometry, with a human breast cancer cell line (MDA-MB-231) as a positive control. Representative frequency distribution curves of logarithmic fluorescent intensity for each cell line are shown (EpCAM antibodies: pink solid curves; rabbit serum or goat gamma globulin controls: gray solid curves) (*n* ≥ 3 independent experiments).

**Figure 4:**
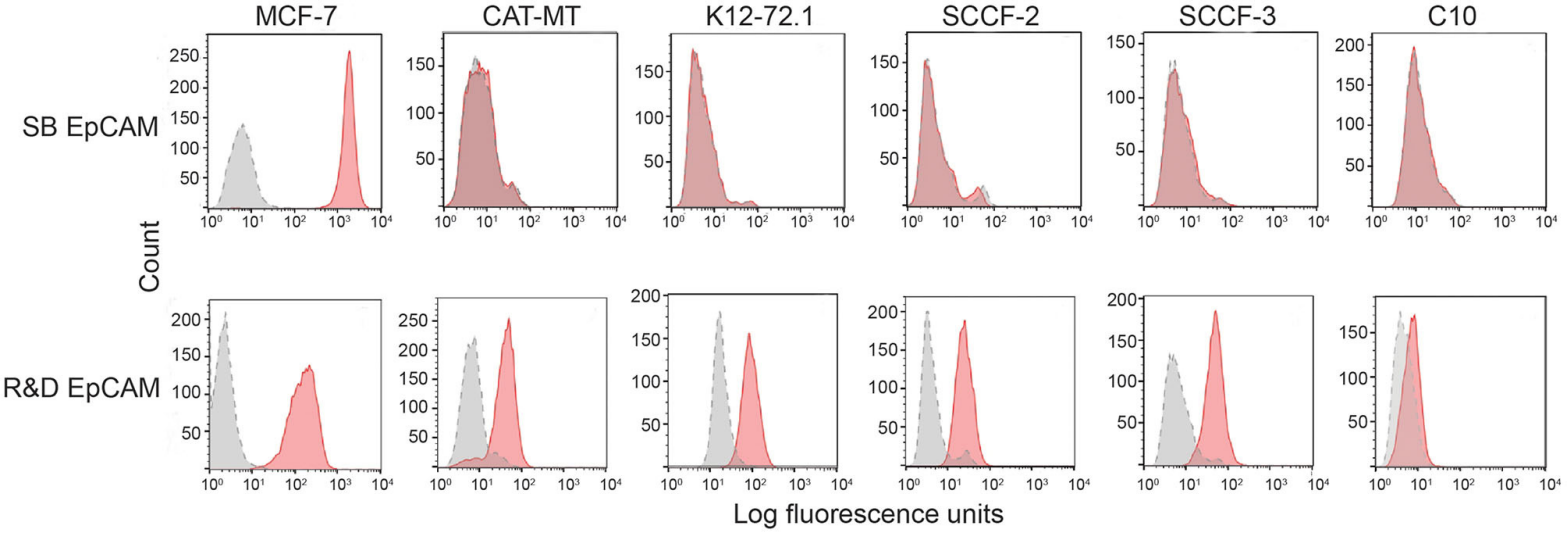
Screening of feline mammary carcinoma (CAT-MT, K12-72.1), oral squamous cell carcinoma (SCCF-2, SCCF-3), and an injection site sarcoma-derived (C10) cell lines with the rabbit monoclonal (SB EpCAM) and goat polyclonal (R&D EpCAM) anti-human EpCAM antibodies with flow cytometry. The MCF-7 human breast carcinoma cell line was used as a positive control. Representative frequency distribution curves of logarithmic fluorescent intensity for each cell line are shown (*n* ≥ 3 independent experiments). Only the R&D EpCAM antibody showed consistent binding to the feline epithelial cell lines but not the sarcoma cell line (EpCAM antibodies: pink solid curves; rabbit serum or goat gamma globulin controls: gray solid curves with dotted line).

We first tested the two antibodies against the human breast carcinoma positive control cell lines, MCF-7 and MDA-MDA-231, as both cell lines should express EpCAM, with MCF-7 being the higher expressing cell line (23). The expected expression of EpCAM was confirmed with both antibodies (Figure 2). We next tested these antibodies for surface reactivity against the feline cell lines, using the same human breast cancer cells, as positive controls. All feline cell lines were tested with both antibodies, with the exception of FMEC and NLFK, which were not tested with the SB EpCAM antibody. Positive staining was seen with the goat polyclonal R&D EpCAM antibody on the feline normal and neoplastic mammary cell lines and the oropharyngeal squamous cell carcinoma cell lines, whereas no staining was evident on the NLFK normal renal epithelial cell line (Figures 3, 4 and Table 2). The C10 injection site sarcoma cell line showed a weak shift in fluorescent intensity in individual experiments that was not considered a true positive result (Figure 4 and Table 2). No positive staining was seen with the rabbit monoclonal SB EpCAM on any of the tested feline tumor cells (Figure 4). Based on the flow cytometric results, the goat polyclonal R&D EpCAM antibody was used for the competitive binding and IHC experiments.

**Table 2:** Delta median fluorescent intensity (Delta MFI; mean ± SD for Gaussian and median and range for non-Gaussian data) of staining of a goat polyclonal anti-EpCAM antibody (R&D EpCAM) on feline mammary carcinoma, squamous cell carcinoma and fibrosarcoma cell lines as assessed by flow cytometry.

**Cell line**	**Origin**	**Delta MFI** ** mean ± SD** ** median (range)**
MCF-7	Human mammary carcinoma	470 (147–505)
CAT-MT	Feline mammary carcinoma	35 ± 22
K12-72.1	Feline mammary carcinoma	56 ± 30
SCCF-2	Feline squamous cell carcinoma	34 ± 22
SCCF-3	Feline squamous cell carcinoma	62 ± 26
C10	Feline fibrosarcoma	2 ± 1

*The MCF-7 human breast carcinoma cell line was used as a positive control for EpCAM staining (n = 4–6 independent experiments per cell line). Delta MFI = MFI of antibody-labeled tumor cells minus MFI of relevant negative control-labeled tumor cells*.

### Competitive Binding of the R&D EpCAM Antibody With Recombinant Human EpCAM on Flow Cytometric Analysis

**Figure 5:**
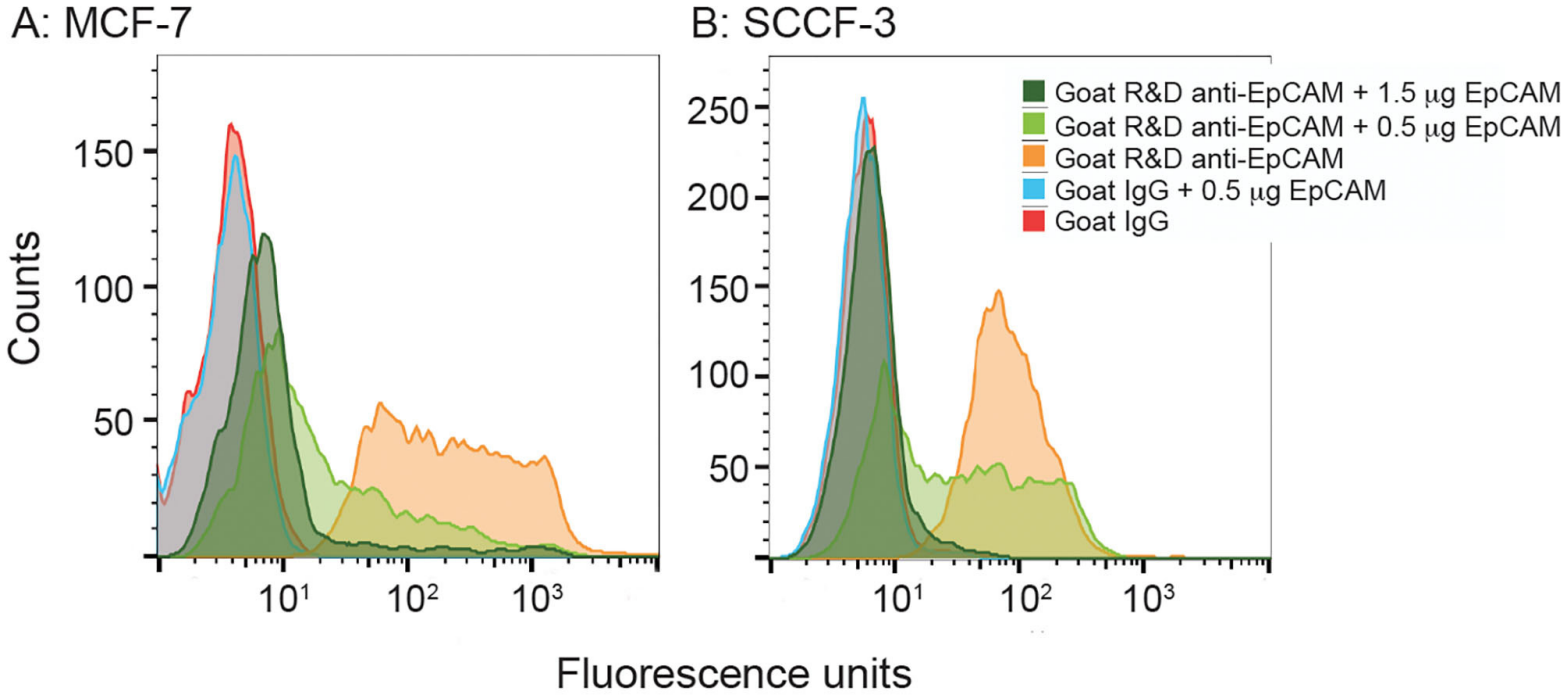
Recombinant human EpCAM competes with tumor cells for binding to the R&D goat polyclonal anti-EpCAM antibody on flow cytometric analysis. Addition of 0.5 or 1.5 μg recombinant EpCAM caused a dose-dependent decrease in binding of the R&D EpCAM antibody to the MCF-7 human breast carcinoma cell line **(A)**. Exposure to 0.5 μg of recombinant protein decreased binding, whereas the higher concentration of 1.5 μg abolished binding, of the R&D EpCAM antibody to the feline oral squamous cell carcinoma cell line, SCCF-3 **(B)**. The recombinant protein did not influence background fluorescence of the goat gamma globulin control for either cell line.

We then used recombinant human EpCAM to competitively block binding of the goat polyclonal R&D EpCAM antibody on feline tumor cell lines. For this purpose, we only tested the SCCF-3 feline oropharynageal squamous cell carcinoma cell line, because this line gave the highest positive staining of the tested feline tumor lines, based on median log fluorescence (Figure 4 and Table 2). The MCF-7 human breast carcinoma cells served as a positive control. We found that addition of 0.5 and 1.5 μg of recombinant EpCAM reduced and abolished R&D EpCAM labeling of the SCCF-3 feline tumor cells, respectively, whereas staining was reduced but not completely inhibited on the higher-expressing MCF-7 cells (Figure 5). This data supports that the goat polyclonal R&D EpCAM antibody is detecting EpCAM on the feline tumor cells.

### Immunohistochemical Detection of EpCAM in Normal and Neoplastic Feline Tissues With the R&D EpCAM Antibody

**Figure 6:**
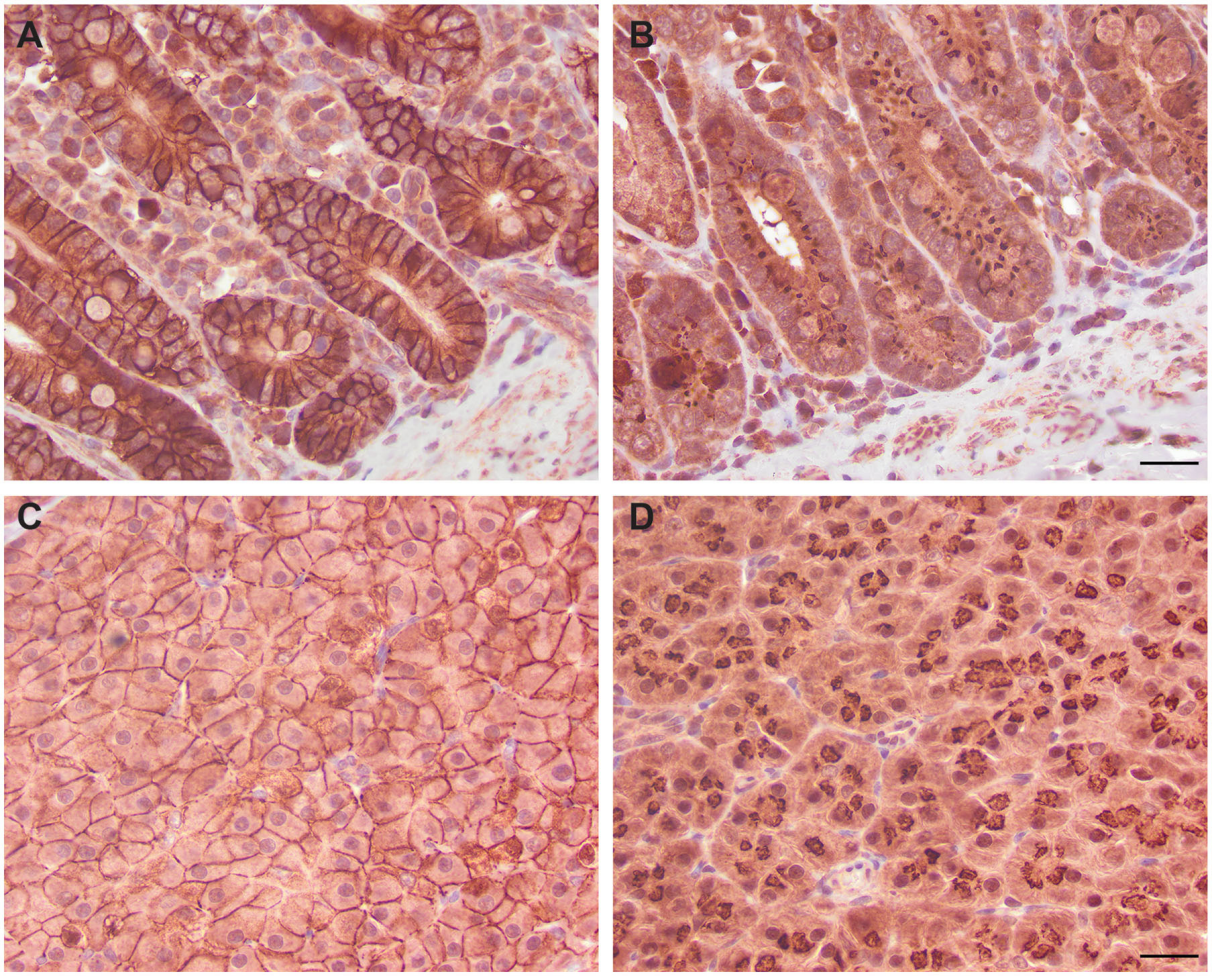
Immunohistochemical staining of duodenum **(A,B)** and pancreatic epithelium **(C,D)** with the R&D goat polyclonal EpCAM antibody **(A,C)** and a goat IgG control **(B,D)** using an initial non-optimized staining protocol on a feline tissue array (DAB chromogen). Positive membranous staining, outlining the intercellular junctions of epithelial cells, is evident with the R&D EpCAM antibody in the duodenum **(A)** and pancreas **(C)**. In the pancreas, both acinar **(C)** and ductular epithelium (not shown) showed similar membrane staining. Substantial background cytoplasmic staining was evident with the negative goat IgG control, but staining of the cell membranes was lacking in either the duodenum **(B)** or pancreas **(D)**. Scale bar = 20 μm.

**Figure 7:**
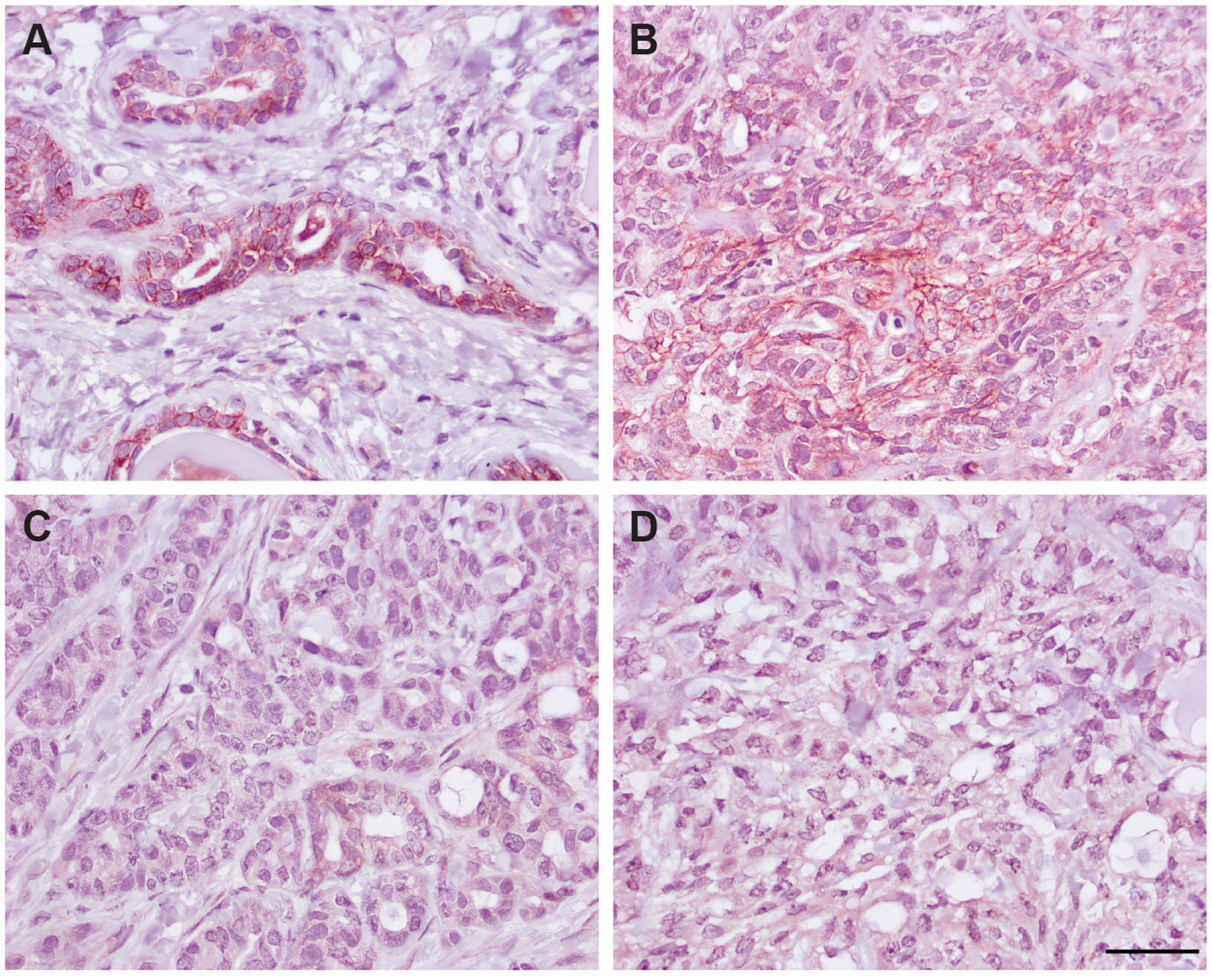
Immunohistochemical staining of a grade III mammary tubular carcinoma in a cat with the R&D goat polyclonal anti-EpCAM antibody using the optimized protocol (DAB chromogen). Several areas of the section contained unaffected normal mammary epithelium, which showed positive cell membrane staining **(A)**. Only small sections of the tumor showed positive membrane staining **(B)**, whereas much of the tumor did not stain with the antibody **(C)**. Minimal staining was seen with the goat IgG control in the normal mammary epithelium (not shown) or throughout the mammary tumor (**D**, scale bar = 20 μm).

We next determined if the R&D EpCAM antibody was detecting a protein with the expected expression pattern of EpCAM in feline tissues. We thus tested the antibody on formalin-fixed, paraffin-embedded feline tissue arrays. We observed positive membrane staining, outlining cell junctions in pancreatic acinar and ductular epithelium and intestinal epithelium, including duodenum (Figure 6), jejunum and colon, but not in the other tested tissues on the array. The lack of IHC staining in renal epithelium in the tissue array fits with the lack of labeling of NLFK renal epithelial cells with the same antibody in flow cytometric testing (Figure 3), indicating that the antibody only detects feline EpCAM in select epithelium. Due to the dark non-specific background staining with the initial IHC protocol used to screen the array, we then used an optimized protocol to test for EpCAM expression in formalin-fixed, paraffin-embedded sections of normal feline oral tissue, two feline mammary carcinomas, one with unaffected normal mammary and cutaneous epithelium, and an oral squamous cell carcinoma. We found that the unaffected normal mammary epithelium in the section of the grade III mammary tubular carcinoma had positive membranous staining with the R&D EpCAM antibody, but variable expression was seen within the mammary carcinoma in the same section (Figure 7). The grade II mammary tubulopapillary carcinoma also showed non-uniform staining for EpCAM in the neoplastic epithelial cells (Supplementary Figure 1). We did not observe any positive IHC staining for EpCAM in the oral tissue from a healthy cat, normal cutaneous epithelium in the section of the mammary tumor, or the tested oral squamous cell carcinoma (Supplementary Figure 2). We also found batch to batch variation in staining intensity with the antibody, necessitating low antibody dilutions (1:10) in the final optimized protocol.

### Static Adhesion Assays With the R&D anti-EpCAM Antibody

**Figure 8:**
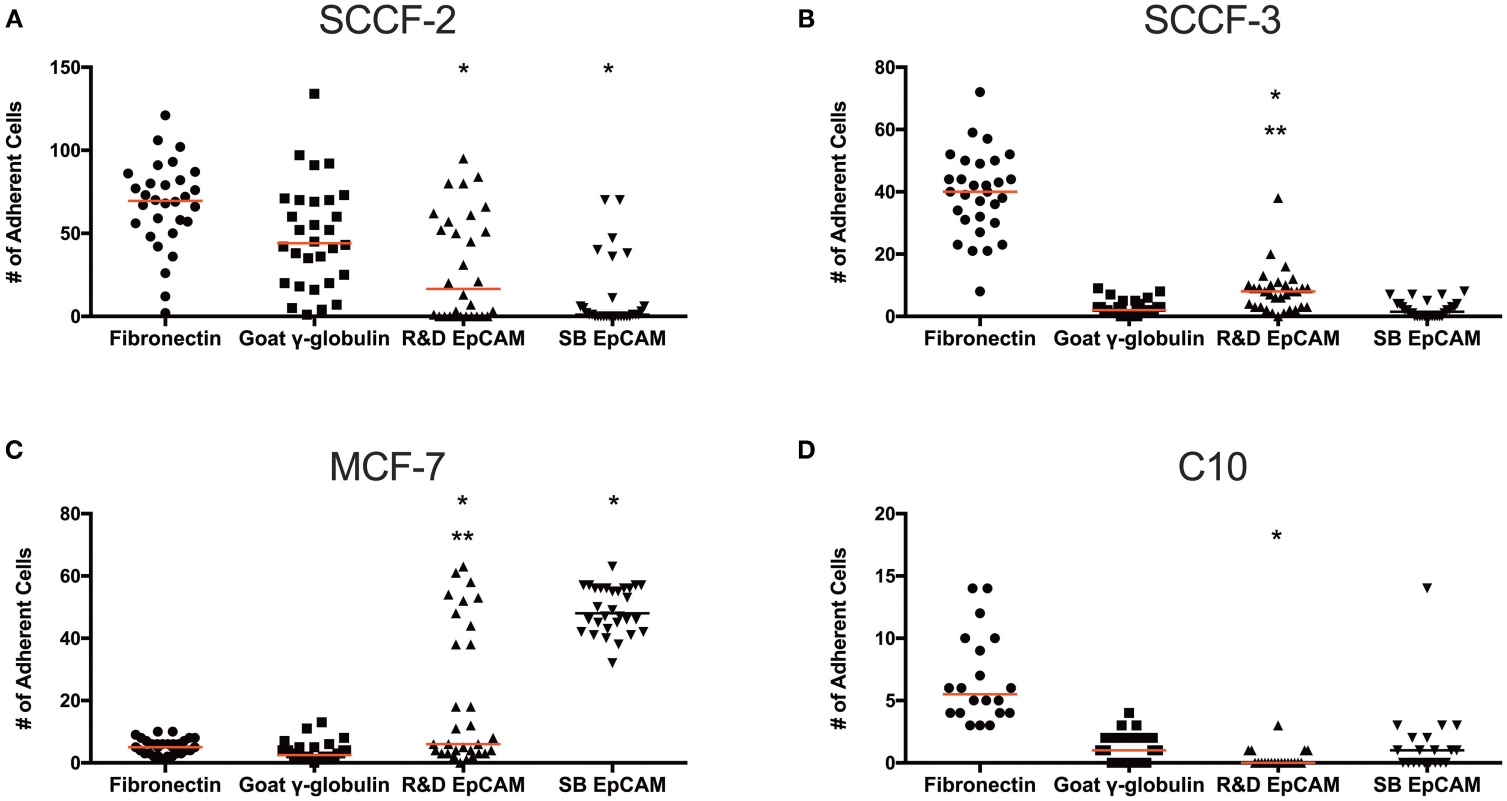
Binding of feline oral squamous cell carcinoma cell lines to the R&D goat polyclonal anti-EpCAM antibody-coated coverslips using a static adhesion assay. Binding was determined by a blinded observer, who counted fluorescent nuclei in 10 randomly photographed fields per coverslip, after cells were allowed to bind for 1 h, followed by washing and fluorescent labeling of nuclei. Individual data points are shown with medians (red line). We tested the feline oral squamous cell carcinoma cell lines (SCCF-2, **A**, and SCCF-3, **B**) with the MCF-7 human mammary carcinoma **(C)** and feline C10 injection site sarcoma **(D)** cell lines as positive and negative cell controls, respectively, on R&D EpCAM-coated coverslips (triangles), with goat gamma globulin (γ-globulin; squares) and the rabbit monoclonal SB EpCAM antibody (inverted triangle) as negative coating controls for the feline cell lines. The SB EpCAM antibody was used as a positive control for the MCF-7 cells. Fibronectin was used as an integrin-mediated binding control (circles). Note that there is a different scale on the Y-axis for each cell line. *p < 0.05 comparing R&D EpCAM- to goat γ-globulin-coated coverslips. **p < 0.05 comparing R&D EpCAM- to SB EpCAM-coated coverslips.

To test if the R&D EpCAM antibody would be suitable for flow-based capture of CTCs in cats, we used a “best case scenario” static adhesion assay, in which cells settle by gravity then bind to antibody-coated coverslips vs. attempting cell capture under fluid flow. We tested the feline oropharyngeal squamous cell carcinoma cell lines, SCCF-2 and SCCF-3, as our model cells with R&D EpCAM-coated coverslips, using the rabbit monoclonal SB EpCAM antibody- and goat γ-globulin-coated coverslips as negative controls and fibronectin-coated coverslips as a control for integrin-mediated adhesion. The MCF-7 human breast carcinoma and the C10 feline injection site sarcoma cell lines were used as positive and negative cell controls, respectively, for EpCAM-mediated adhesion. We found that R&D EpCAM antibody-coated coverslips bound fewer SCCF-2 than the γ-globulin-coated control, with non-specific binding evident with the negative control SB EpCAM antibody-coated coverslips. Significantly more SCCF-3 cells bound to R&D EpCAM antibody- vs. SB EpCAM- and γ-globulin-coated coverslips. In contrast, significantly more MCF-7 cells bound to R&D and SB EpCAM antibody-coated coverslips vs. the γ-globulin-coated control. The C10 feline injection site sarcoma cell line only consistently bound to the fibronectin-coated coverslips (Figure 8).

## Discussion

Our results indicate that only one of the two tested antibodies, the goat polyclonal R&D anti-EpCAM antibody, detected a membrane-expressed protein on tissue-cultured feline cell lines derived from normal and neoplastic mammary epithelium and neoplastic squamous cells from the oropharyngeal mucosa with flow cytometry. The same antibody recognized a membrane-associated protein in normal mammary, intestinal (duodenum, jejunum, colon) and pancreatic acinar and ductular epithelium in formalin-fixed tissues from cats. However, the antibody demonstrated weak affinity for the protein, with lot-to-lot variability, and was unable to “capture” positively-labeled feline oropharyngeal squamous cell carcinoma cells in static adhesion assays. Thus, the two anti-human EpCAM antibodies tested in this study would not be worthwhile pursuing for immunologic-based detection of CTCs in the blood of cats and generation of feline-specific antibodies may be required for these “liquid biopsies.”

Polyclonal antibodies, such as the goat-based R&D antibody against human EpCAM, are more likely to cross-react with the protein in other species vs. a monoclonal antibody that may detect a species-unique epitope on the target protein. Thus, it is not surprising, albeit disappointing, that the monoclonal SB antibody did not detect EpCAM on feline cells. Other commercially available antibodies may be cross-reactive with the feline protein on flow cytometric analysis, however testing different clones becomes prohibitively expensive, particularly with a less than certain outcome. We only tested the selected antibodies with flow cytometry for the specific purpose of using flow-based techniques for detecting CTCs in the future. It is possible, however, that the SB EpCAM antibody or other antibodies would have worked with other methods, such as immunoblotting or IHC.

Binding of the goat polyclonal R&D EpCAM antibody to the highest expressing feline oropharyngeal squamous cell carcinoma and human MCF-7 breast carcinoma cell lines was competitively inhibited by addition of recombinant human EpCAM. This data supports that the antibody was detecting the feline equivalent of EpCAM vs. non-specifically binding to another protein. Additional support came from the lack of positive staining of an injection site sarcoma cell line by flow cytometry and positive membrane staining of normal feline pancreatic, intestinal and mammary epithelium on tissue sections, which is the expected pattern of expression for this transmembrane protein (9). In contrast to human tissue (9, 12), positive staining was not identified in other epithelial tissues of the array, including kidney (loop of Henle, distal and collecting tubules), lung, and thyroid. This could reflect true species differences in EpCAM expression, technical problems, such as the strong background obscuring weak reactions, low antibody avidity for the feline protein (low dilutions were required for protein detection in formalin-fixed tissue), and potential cross-reaction of the antibody with a non-EpCAM membrane-associated protein in feline tissues. Distinguishing between these possibilities will rely on other techniques, such as documentation of RNA expression (e.g., *in situ* hybridization), or repeat IHC staining with a feline-specific or higher avidity cross-reactive anti-EpCAM antibody.

EpCAM is not expressed on all epithelial tissue and is unlikely to be a universal marker of epithelial-derived tumors. For instance, only patchy staining is observed in gastric mucosa and squamous epithelium of the skin and the proximal convoluted tubules and glomeruli in the kidney are negative for EpCAM on IHC staining of human tissue (9, 12). In this study, we found that pancreatic and intestinal epithelium was strongly labeled with the antibody in a normal feline tissue array, suggesting that EpCAM may be a potential IHC marker for epithelial tumors derived from these tissues. However, EpCAM may be upregulated in a tumor arising from epithelium that normally lacks expression of the protein, such as cutaneous squamous cell carcinomas in humans (8, 12). We found that two feline oropharyngeal squamous cell carcinoma cell lines stained with the R&D EpCAM antibody on flow cytometry but the protein was not detected in a formalin-fixed tissue section of an oral tumor from a cat with IHC using the same antibody. It is possible that expression on the cell lines is a consequence of tissue culture or that the antigenic epitopes recognized by the antibody were cleaved during processing for IHC (33). However, we only tested for EpCAM staining in one feline oropharyngeal squamous cell carcinoma and expression can be variable in human patients with this tumor, ranging from 62–86% of tested patients for cervical and esophageal squamous cell carcinomas, with lower expression in the former (7). Similarly, EpCAM expression ranges from 45–100% in different breast carcinomas in human patients and expression can be uniform or heterogeneous in various epithelial tumors (7, 9). Non-uniform expression was also seen in the two feline mammary carcinomas evaluated in this study. It is possible that EpCAM expression signals more aggressive behavior in epithelial tumors. There is a higher frequency of EpCAM expression in metastatic vs. primary tumors and ductular compared to lobular carcinomas in human breast cancer patients (7). In addition, higher EpCAM expression on CTCs could be a marker of more aggressive tumors (7, 8, 15). This theory remains to be tested with IHC staining of different grades of mammary carcinomas and primary and metastatic tumors in cats. However, due to the low antibody dilutions required for IHC and the non-specific background, even after optimization of the IHC staining protocol, additional tumors were not tested for EpCAM staining in this study, which is a study limitation. Future testing with a higher avidity antibody or a specific anti-feline EpCAM antibody is needed to more fully determine the expression of EpCAM in normal and neoplastic feline tissues.

The monoclonal SB EpCAM antibody yielded more consistent adhesion than the polyclonal R&D EpCAM with the high EpCAM-expressing MCF-7 human breast carcinoma cell line, further illustrating that the latter antibody would not be optimal for CTC detection. Similarly, the polyclonal R&D EpCAM antibody showed substantial variability in capturing the SCCF-3 feline oropharyngeal squamous cell carcinoma cell line under static conditions. Variability of antibody-based adhesion in this study may be partly explained by random orientation of the antibody on the coverslips, but this should similarly affect binding of the MCF-7 cells to both the SB and R&D EpCAM antibodies. The SCCF-2 cell line showed substantial ligand-independent variability in adhesion, suggesting tumor-specific differences in adhesiveness. We only tested the feline oropharyngeal squamous cell carcinoma cell lines in the static adhesion assay and not the feline mammary carcinoma cell lines. It is possible that different adhesion patterns would have been seen with the latter cell lines. EpCAM can be downregulated during epithelial-to-mesenchymal transition, which can precede transmigration of tumor cells from interstitial tissues to the intravascular space, limiting CTC detection. Therefore, methods to capture CTCs using antibody cocktails against various markers have been developed (3, 17, 34). Evaluation of antibodies against other epithelial or tumor-based markers, such as Mucin 1 and epidermal growth factor receptor (35, 36), for detection of epithelial-derived CTCs in cats would be worthwhile.

## Data Availability Statement

The raw data supporting the conclusions of this article will be made available from the authors, upon request and without reservation.

## Ethics Statement

Protocols to collect tissues prospectively from cats were approved by the Institutional Animal Care and Use Committee, Cornell University (#2005-0151).

## Author Contributions

CH performed the vast majority of the experiments. LD performed the immunohistochemical staining on the arrays and tumor sections. HS was the blinded observer counting cells in the static adhesion assay. CW performed flow cytometric analysis and the adhesion assays. TS devised this study and performed the competitive binding experiments and a few flow cytometric assays of the tumor cells. All authors contributed to the article and approved the submitted version.

## Conflict of Interest

The authors declare that the research was conducted in the absence of any commercial or financial relationships that could be construed as a potential conflict of interest.
