# Supplementary material for: Detection of Epithelial Cell Adhesion Molecule in Feline Normal and Tumor Cell Lines and Tissues With Selected Commercial Anti-human EpCAM Antibodies
Source: Front Vet Sci. 2021 Feb 4;8:622189. doi: 10.3389/fvets.2021.622189 (PMC7890202; doi:10.3389/fvets.2021.622189)

Supplemental Figure 1: Immunohistochemical staining for EpCAM in formalin-fixed paraffin-embedded histologic sections of a grade II tubulopapillary mammary carcinoma from a cat using a goat polyclonal R&D anti-EpCAM antibody (DAB chromogen). Staining at intercellular junctions is present in surface and deep neoplastic epithelial cells (arrows) with the polyclonal R&D anti-EpCAM antibody (**A, C**) but not the goat IgG control (**B, D**), despite non-specific background staining with the latter. Not all of the tumor showed positive staining reactions (**A, B**: Scale bar = 50 μm; **C, D**: Scale bar = 20 μm).

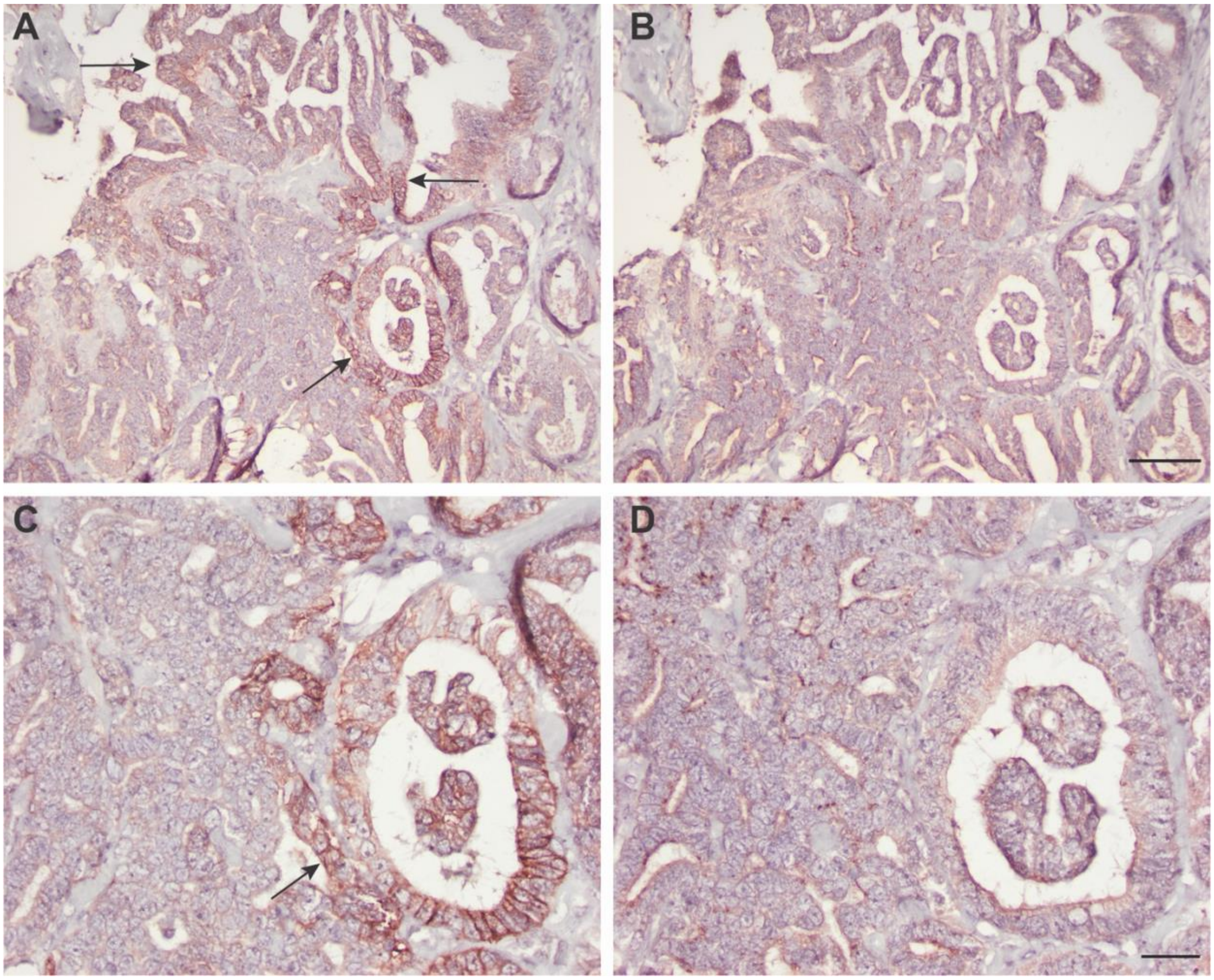

Supplemental Figure 2: Immunohistochemical staining for EpCAM in normal feline cutaneous and oral tissue and an oropharyngeal squamous cell carcinoma using a goat polyclonal R&D anti-EpCAM antibody (**A-B**: DAB chromogen, **C-F**: Nova red chromogen). No membrane staining is evident in the epithelial cells of the skin in a section from a grade III mammary tubular carcinoma in a cat that contained normal cutaneous tissue with the anti-EpCAM antibody (**A**) or goat IgG control (**B**) (scale bar = 50 μm). Similarly, normal gingival mucosa (**C**) and overlying normal gingival tissue and neoplastic epithelial cells in an oropharyngeal squamous cell carcinoma (**E**) were negative when stained with the R&D EpCAM antibody, as were the corresponding goat IgG controls (**D**, **F**) (scale bars = 50 μm).

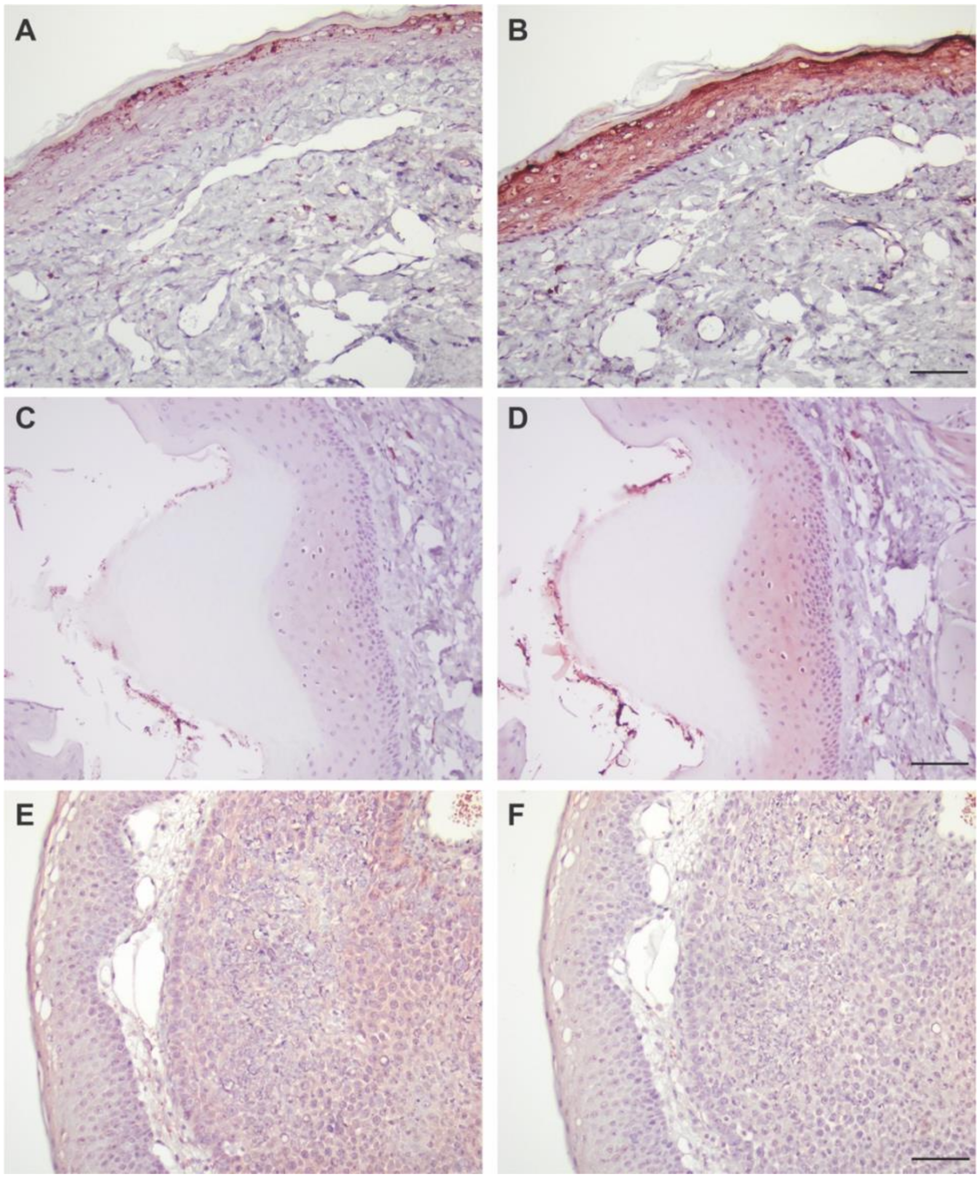

Supplement: Supplementary file 1 [file Data_Sheet_1.pdf]
